# The anti-ethylene growth regulator silver thiosulfate (STS) increases flower production and longevity in cassava (*Manihot esculenta* Crantz)

**DOI:** 10.1007/s10725-019-00542-x

**Published:** 2019-09-20

**Authors:** Peter T. Hyde, Xian Guan, Viviane Abreu, Tim L. Setter

**Affiliations:** Section of Soil and Crop Sciences, School of Integrative Plant Science, Cornell University, 517 Bradfield Hall, Ithaca, NY USA

**Keywords:** Flowering, Ethylene, PGR, STS, Silver thiosulfate, Cassava

## Abstract

Cassava, which produces edible starchy roots, is an important staple food for hundreds of millions of people in the tropics. Breeding of cassava is hampered by its poor flower production, flower abortion, and lack of reproductive prolificacy. The current work determined that ethylene signalling affects floral development in cassava and that the anti-ethylene plant growth regulator silver thiosulfate (STS) mitigates the effects of ethylene on flower development. STS did not affect the timing of flower initiation, but improved early inflorescence and flower development as well as flower longevity such that flower numbers were increased. STS did not affect shoot and storage root growth. Studies of silver accumulation and treatment localization support the hypothesis that the beneficial effects of STS are confined to tissues of the shoot apex. The most effective timing of application was before inflorescence appearance extending to post-flower appearance. Based on this work a recommended protocol for STS use was developed. This work has the potential to improve methods for enhancing cassava flower development in breeding nurseries and thereby synchronize flowering of desired parents and enable the production of abundant progeny of desired crosses.

**Electronic supplementary material:**

The online version of this article (10.1007/s10725-019-00542-x) contains supplementary material, which is available to authorized users.

## Introduction

Cassava (*Manihot esculenta* Crantz) is a crop grown in tropical regions for its high-starch storage roots. It ranks as the fourth largest source of energy in human diets in the tropics, after maize (*Zea mays*), rice (*Oryza sativa*) and wheat (*Triticum aestivum*). In sub-Saharan Africa, where it is valued for its stability of production in stressful environments, over 500 million people depend on cassava for food security (Ceballos et al. 2010; Jarvis et al. 2012; Rosenthal and Ort 2012). Continual crop improvement through management and breeding is needed to increase yields, broaden production uses, and alleviate vulnerability to abiotic and biotic stresses. Conventional breeding methods and the recently developed breeding system involving genomic selection (Wolfe et al. 2017) hold promise, but to be successful they require relatively rapid and synchronous flowering to speed up the breeding cycle (Ceballos et al. 2015; Heffner et al. 2009; Wolfe et al. 2017). An obstacle to cassava breeding is that many genotypes with valuable agronomic characteristics flower extremely late, have poor flower development, and abort before viable seed is produced (Adeyemo et al. 2017, 2018; Ceballos et al. 2017). In the past, cassava flowering was not a priority in selection because the harvested part is the storage root, and there is evidence of a negative correlation between storage root yield and flower initiation as indicated by branch number (Tan and Cock 1979). Floral initiation occurs at the apical meristem and stimulates forking (production of 2–4 branches), thereby producing an axil where the flower structure develops; such forking occurs periodically at the shoot apical meristem and creates a series of sympodia (tier 1, tier 2, etc.). Cassava typically does not produce viable flowers at its first forking event and some genotypes are not known to flower at all (Adeyemo et al. 2018; Alves 2002; Perera et al. 2013). Further exacerbating the problem, each inflorescence produces only a small number of female flowers (Perera et al. 2013) and pollination typically produces only 1–2 seeds per flower (Ceballos et al. 2010). The development of methods to promote earlier, and more abundant flowering, with better flower longevity and viability has the potential to facilitate faster cycles of breeding and more rapid progress.

Endogenous plant hormones are among the most important factors that regulate flower and fruit development, maturation and senescence. Possible uses of plant growth regulators (PGRs) to regulate plant reproductive development have been researched in many plant species (Rademacher 2015). As a starting point for investigations of flower-enhancing treatments in cassava, investigators associated with the Nextgen Cassava project (www.nextgencassava.org), an international cassava breeding collaboration, screened several plant growth regulators for their effects on flowering (Abah et al. 2016; Abubakar et al. 2016; Hyde et al. 2016). Among the PGR candidates, including cytokinins, auxins, gibberellins, anti-GA, jasmonic acid, and salicylic acid, the most promising results in glasshouse studies were obtained with the anti-ethylene PGR, silver thiosulfate (STS) (Supplementary Table S1; Hyde et al. 2016). Ethylene regulates reproductive development in several species by manipulating floral induction (Achard et al. 2007; Kesy et al. 2008), hastening abscission and senescence of flowers (Iannetta et al. 2006; Onozaki et al. 2018; Serek et al. 2006; van Doorn 2002), enhancing fruit ripening (Barry and Giovannoni 2007), and decreasing fruit set (Martínez et al. 2013). Plant growth regulators have been used to block the ethylene signalling pathway at several steps (Serek et al. 2006). Various inhibitors of key enzymes in ethylene synthesis have been used to decrease the rate of ethylene formation (Kosugi et al. 2014; Serek et al. 2006). Alternatively, chemical agents such as 1-methylcyclopropene (1-MCP) and silver (Ag^+^), which bind to the ethylene receptor and thereby block its signal transduction, have been used to prevent ethylene signalling (Beyer 1976; Serek et al. 2006; Serek et al. 2015; Veen 1983; Veen and van de Geijn 1978). However, preliminary trials with 1-MCP were not effective in improving flowering in cassava (Supplementary Table S1, worksheets PGR 4 and 5). To improve its uptake and transport, Ag^+^ is applied in the complex silver-thiosulfate (STS) (Veen and van de Geijn 1978). Thus, whereas 1-MCP is a gas which must be delivered to the target tissue in a confined atmosphere or with an encapsulation delivery system, STS has a long residence time within the plant and it is freely transported cell-to-cell and via the vascular system to the desired target tissue where it is available to continuously bind to new ethylene receptors as the plant grows (Serek et al. 2006; Veen and van de Geijn 1978).

The objectives of the current work were (a) to determine the extent to which ethylene signaling affects floral development in cassava and (b) to determine the most effective parameters for using STS as an anti-ethylene plant growth regular (PGR) to mitigate the effects of ethylene. Our findings indicate that ethylene does not affect the timing of flower initiation, but inhibits early inflorescence and flower development as well as flower longevity, and STS is effective in reversing these effects. Ethylene did not affect vegetative and storage root growth. Our studies of silver accumulation and treatment localization support the hypothesis that shoot apex tissues are the target for beneficial effects of STS.

## Materials and methods

### Plant material

Four cassava genotypes were used for the STS studies. TMSI980002 (also known as TMS IBA980002, TMS I980002 and IBA980002) and TMEB 419 (also known as TME 419) were obtained from the International Institute of Tropical Agriculture (IITA), Ibadan, Nigeria; NASE 3 (also known as TMS 30572) and TME 204 were obtained from the National Crop Resources Research Institute (NaCRRI), Namulonge, Uganda. For the STS-ethephon experiment, the cassava genotypes FT2, FT11, FT13, and FT17, were obtained from the Center for International Agriculture of the Tropics (CIAT), Palmira, Columbia. These lines were the cassava genotype 60444 transformed to overexpress *Arabidopsis thaliana* FT (Adeyemo et al. 2017).

### Growth conditions

Cassava stem sections (stakes) ca. 15 cm long, 2.5 cm in diameter, cut from the bottom 1 m of plants 6 months old, were planted into 11 L pots containing soil-less growing media, consisting of 62% (v/v) peat moss (Lambert Peat Moss LG, Lambert Peat Moss Inc., Quebec, Canada), 22% (v/v) vermiculite (Coarse Vermiculite, Whittemore Company Inc., Lawrence MA 01843, USA), 11% (v/v) perlite (Super Coarse Perlite, Whittemore Company Inc.), 2.2% (w/v) dolomitic limestone (Microfine Dolomite, The National Lime and Stone Co., Findlay Ohio, 45840, USA), 0.1% (w/v) wetting agent (AquaGro 2000G, Aquatrols, Paulsboro, NJ 08066, USA), and 2.2% (w/v) 10-5-10 Jacks Pro Media mix plus III (J.R. Peters, Inc., Allentown, Pennsylvania, USA).

These plants were grown in a glass house with supplemental heat as needed to attain a temperature of 30 °C from 6:00 until 18:00 (day) and 25 °C from 18:00 until 06:00 (night). Supplemental lighting from 400 Watt metal halide lamps spaced at 80 × 190 cm (PX-MPS400/7 K, PlantMax, 1000Bulbs Co., Garland, TX, USA) was provided between 06:00 am and 20:00 pm when solar photosynthetic (400–700 nm) photon flux density was < 500 μmol m^−2^ s^−1^.

### Plant growth regulator (PGR) materials and application

Ethephon (2-chloroethylphosphonic acid; 2SL, Makhteshim Agan of North America, Inc., Raleigh, NC 27604, USA) solution was prepared with 500 ppm (w/v) ethephon. Silver thiosulfate (STS) was prepared as follows: A 0.1 mol/L solution of silver nitrate (Sigma-Aldrich, St. Louis, MO, USA) was slowly mixed into a 0.1 mol/L sodium thiosulfate (Sigma-Aldrich) at a 1:4 silver nitrate to sodium thiosulfate ratio by volume. The resulting STS stock solution, with 20 mmol Ag^+^/L, was diluted with reverse osmosis (RO) purified water to the desired concentrations described below and in figure and table legends, where the reported mM concentration of STS refers to the Ag^+^ concentration contained therein. Each solution contained 0.1% (v/v) Tween 20 (Sigma-Aldrich, PO Box 14508 St. Louis, MO 63178, USA). For all STS experiments except the localization and timing experiment (described below), a 100 mL treatment of each was applied by spraying all leaves using a 1.5-gallon Solo® 450 series sprayer (Solo, 5100 Chestnut Avenue Newport News, VA 23605) with a 0.14 MPa (21 psi) constant flow valve (item no. 163124, Gempler P.O. Box 5175, Janesville, WI, USA).

### Flower terminology and data collection

All plants were evaluated weekly to determine the time of flower appearance. In the present study, what is botanically considered a cyathium (Perera et al. 2013), is referred to as a flower, and what is botanically a set of cyanthia on modified stem (stalk) arising from a shoot branch-point is referred to as an inflorescence. If flowers were present, the number of flowers (diameter ≥ 2 mm) were counted, the number of flowers that had reached anthesis (open flowers) and the length of the floral inflorescence was measured from the point of attachment on the stem fork to the tip of the terminal flower (inflorescence length). Using such data, obtained weekly for each plant, we determined (1) the number of flowers on a plant at a given time point, including immature buds and flowers that matured to anthesis (flowers), (2) the greatest number of flower buds across all time points (maximum flower count), (3) the duration over which flowers were present on a given plant before they abscised (flower retention), and (4) the sum of flower buds across all time points (flower integral).

### Measuring the rate of ethylene production

Two lobes of leaf tissue from the second most recently matured leaf were cut from each plant, sealed with a serum stopper in a 30-mL test tube, and incubated at 25 °C for 24 h. After 24 h, one mL of the gas inside the test tube was sampled and ethylene concentration was measured using a gas chromatograph (Buck Scientific, Model 310, Norwalk CT, USA) fitted with an alumina column with a flame ionization detector. Leaves were then dried and quantity of ethylene produced per gram of dried tissue and per incubation time was calculated.

### Measuring silver in leaves

Two weeks after STS was sprayed onto mature leaves, newly formed leaves that did not receive direct spray were sampled. Silver was measured in these samples by the Cornell Nutrient Analysis Lab (CNAL, Bradfield Hall, Ithaca, NY 14853, USA) using inductively coupled plasma–atomic emission spectrometry (ICP-AES, Spectro Analytical instruments Inc., Kleve, Germany), and silver per g dried leaf tissue was calculated.

### Statistical analysis

For the STS Experiment 1 and STS dosage experiment, a randomized complete block design was used, with the number of blocks (batches of plants) and within‐block replicates described below in the sections describing each experiment. A mixed model ANOVA was conducted with the following sources of variation: treatment effect, genotype effect, block effect, and genotype by treatment interaction. The analyses were conducted in R studio (R Core Team 2018). The lsmeans package (Lenth 2016) was used for post‐hoc analysis of pairwise and multiple mean comparisons, as appropriate for each experiment (see Table and Figure legends). For pairwise comparisons in STS Experiment 1, this package uses a Wald type t‐test with degrees of freedom calculated using a Kenward–Roger estimation technique. Multiple comparisons were performed using Tukey’s honest significant difference (HSD) test, or if sample sizes were unequal, using Tukey–Kramer’s HSD. For the experiments testing ethephon × STS, localization of STS treatment to the apical region, and timing of STS treatment, a completely randomized design was used.

### STS experiment 1

The greenhouse temperature was 29.1±4.3 °C (average ± SD) between 06:00 and 18:00 (day) and 21.6±3.7 °C between 18:00 and 06:00 (night). A randomized complete block design was used. Eight plants of each genotype (TMSI980002, NASE 3, TME 204 and TME 419) were assigned to blocks based their initial similarity of height, with the mean height of each block being 26, 47, 58, and 77 cm. A complete set of treatments was randomly assigned within each block. Each block contained two plants of each genotype, each receiving one of the two treatments, either 100 mL of STS with 0.5 mM Ag^+^, 0.1% (w/v) Tween 20 solution (STS treatment) or 100 mL of water, 0.1% (w/v) Tween 20 solution (Control). The first application was applied at 75 days after planting (DAP), before any plants had forked or flowered. Treatments were reapplied at 89 DAP, 103 DAP and 117 DAP.

### STS dosage experiment

The greenhouse temperature averaged 27.4±4.8 °C (day) and 21.8±3.3 °C (night). Twenty plants of each genotype (TMSI980002, NASE 3, TME 204 and TME 419) were used in a randomized complete block design. Four replicates of each genotype × treatment combination were used for a total of 80 plants. Plants were sorted into four blocks of similar height, with the mean height of 46.5 cm, 64.5 cm, 69.1 cm, and 81.8 cm, before treatments were randomly assigned. Treatments were randomly assigned within block and genotype. Treatments consisted of four concentrations of silver thiosulfate (STS); 1.0, 0.5, 0.25 and 0.125 mM of silver thiosulfate with 0.1% (w/v) Tween 20 and a water control with 0.1% (w/v) Tween 20. Plants were sprayed at the following number of days after planting (DAP): 127, 141, 155, 169, 183, 197, 211, 225, 239, and 253. At the conclusion of the experiment, above ground plant material and storage roots greater than 5 mm were dried separately at 55 °C and weighed separately. Total plant dry weight is the sum of the above ground dry weight and the storage root dry weight. Harvest index is the fraction of the total dry weight that is storage root. Root count is the number of storage roots greater than 5 mm.

### STS ethephon experiment

The temperature averaged 30.8±3.3 °C (day) and 24.7±2.8 °C (night). A total of 16 plants were used, four of each genotype. Four different spray treatments were randomly assigned to each genotype and evaluated: (1) water control, (2) two sprays of STS 7 days apart, (3) two STS sprays followed by ethephon, or (4) ethephon alone. STS applications were with 0.5 mM STS at 152 DAP and 159 DAP. Ethephon treatment was 500 ppm (w/v) ethephon at 161 DAP.

### STS localization experiment

The STS treatments (0.25 mM STS) were applied to TMSI980002 plants every 2 weeks for 5 times. Treatment localizations, which were randomly assigned, were: (1) Applying STS to mature leaves only (STS-Leaves); STS was sprayed as described in Experiment 1 except a plastic bag was used to cover the young expanding tissues of the shoot apex to prevent them from being sprayed; Approximately 100 mL of STS solution was applied. (2) Applying STS to the apex only (STS-Apex); Aluminum foil was wrapped around the target apical region to isolate the sprayed region and collect any dripping solution to prevent any liquid from flowing to the mature leaves below. Approximately 10 mL was sprayed. (3) A water control in which 100 mL of water was applied as a general foliage spray.

### STS timing experiment with applications localized to the apical region

Plants were grown to the stage of first tier forking, after which treatments were randomly assigned to four replicate plants of the genotype TMSI980002. STS was administered using a cotton swab (pad) method whereby a piece of unwoven cotton fiber (5 × 4 × 0.3 cm) was soaked with STS solution (10 mL of 0.25 mM STS) then wrapped around the targeted shoot apical region and its expanding tissues. A plastic bag was then put around the cotton swab and tied at the base to maintain high humidity inside the bag. The plastic bag and cotton swabs were removed after 24 h. All treatments were started before the date of flower appearance. Four treatments were applied, each with four weekly applications, differing in the time when STS applications commenced (and concluded): (1) Early (commencing applications at 24–27 days before flowering); (2). Medium Early (starting at 20–23 days before flowering); (3). Late (starting at 13–16 days before flowering; (4). Control (applying water only).

## Results

### STS effects on flower development

**Fig. 1:**
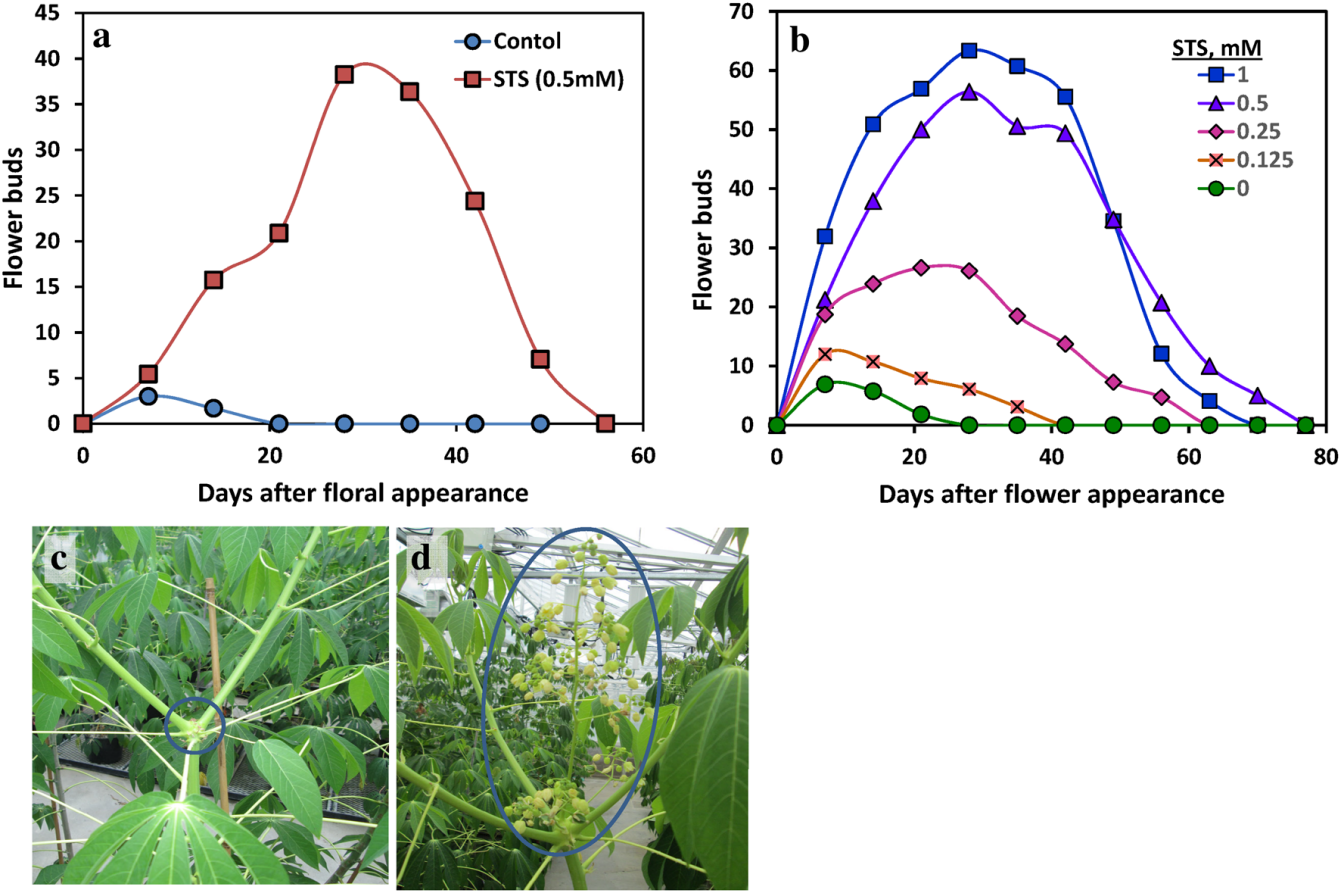
The effect of spray application of STS on floral development; flower counts are for the first tier of flowering and are means of four replicates of four genotypes (TMSI980002, TMEB 419, TME 204, and NASE 3). **a** STS Experiment 1 with 0.5 mM STS foliar spray; **b** STS dosage experiment with the indicated concentration in STS spray; **c** control TMSI980002 plant with the tier 1 branch region encircled where inflorescence development had initiated, then aborted; **d** TMSI980002 plant treated with 0.5 mM STS with tier 1 branch region and inflorescence/flowers encircled

Preliminary trials with a wide range of PGRs, including the cytokinin benzyl adenine, abscisic acid, jasmonic acid, and salicylic acid, indicated that the anti-ethylene agent STS was uniquely effective in improving cassava flowering (Supplementary Table S1; Hyde et al. 2016). We also found 1-MCP to be ineffective in preliminary trials (Supplementary Table S1, worksheets PGR4 and 5). We investigated STS effects on cassava flower production and flower longevity by spraying foliage four times at two-week intervals beginning at 75 days after planting (DAP), before any of the plants had flowered, and continued treatments through the time-frame of first-tier flowering. As shown in the time series averaged over all four genotypes (Fig. 1a), the untreated control plants produced less than ten flower buds, and flowers senesced or abscised in less than 21 days after flowers were first observed. In contrast, STS-treated plants produced over 50 flowers and the longevity of flower production was more than 50 d. STS treatments significantly (P ≤ 0.05) improved floral development by several criteria (Table 1). Flower integral, which is the area under the curve for each plant’s non-senescent flowers over time, as shown in Fig. 1a, provides a measure of flower prolificacy and longevity. All four genotypes responded similarly to STS treatment, as indicated by the absence of Genotype × Treatment interactions (Table 1). Averaged across the five genotypes, STS did not affect the timing of flower appearance, but it significantly (P ≤ 0.05) increased the maximal number of flowers and improved floral longevity by extending the time before floral development ceased and inflorescences senesced. While control flowers senesced 6 days after first appearance, STS treatments extended flower retention more than fivefold to 33 days. Moreover, in the controls, all flowers aborted while still in the bud stage, whereas the plants treated with STS produced fully developed, mature flowers. STS significantly (P ≤ 0.05) extended flower longevity and it increased (P ≤ 0.05) the maximal number of flowers in all four genotypes. Flower integral, a composite measure of maximum number of non-senescent flowers and their longevity, was increased (P ≤ 0.05) by STS in three of the four genotypes.

**Table 1:** The effect of STS on floral development in the genotypes TMSI980002, TMEB419, TMEB204, and NASE-3. STS was applied as a floral spray with 0.5 mM STS

Genotype	Treatment	Age at flower appearance (days)		Maximum flower count		Days of flower retention		Flower integral	
Nase-3	Control	98	NS^§^	2	**	2	***	2	***
	STS	94		46		47		206	
TMEB204	Control	120	*	6	.	9	**	8	NS
	STS	106		41		28		66	
TMEB419	Control	110	NS	4	.	7	**	5	*
	STS	104		39		30		140	
IBA980002	Control	77	NS	3	**	7	***	4	**
	STS	77		60		28		181	
Across all genotypes	Control	101	NS	4	***	6	***	5	***
	STS	95		46		33		148	
ANOVA^¶^									
^¶^Treatment main effect		NS		***		***		***	
Genotype effect		***		NS		NS		NS	
Block effect		*				**		NS	
Genotype × treatment		NS		NS		NS		NS	

The symbols ·,*, **, or *** indicate significance at the 0.1, 0.05, 0.01, and 0.001 probability level, respectively. NS indicates no significant difference

^§^Posthoc pairwise comparisons between treatments were performed using a t-test. There were four replicate blocks

^¶^ANOVA based on a model with STS treatment (T), Genotype (G), Block and GXT interaction effects. Analyses were based on square root transformed data

### STS dosage

We used a geometric series of STS concentrations from 0.125 to 1.000 mM to test the dosage response of STS. The extent of improvement of floral development increased with concentration of STS sprayed (Fig. 1b). While inflorescences aborted, senesced, and abscissed at an early stage in untreated controls such that only a small remnant of the inflorescences remained after a few days (Fig. 1c), STS increased flower numbers and longevity, and the morphology of inflorescences and flowers appeared normal and well developed (Fig. 1d). ANOVA indicated that STS treatments did not significantly affect the age at flowering, but they improved the number of flowers produced, flower retention, and flower integral (Table 2). The significant (P ≤ 0.0001) genotype effect on age at flowering was due to the shorter number of days to flowering (DTF) in TMSI980002 (136 days) than in TME 204 and TME 419 (188 and 170 days, respectively). And the significant (P ≤ 0.05) genotype effect on flower integral was due to the larger value, averaged across STS treatments, in TMSI980002 (296) than in TME 204 and TME 419 (131 and 140, respectively). Averaging across all genotypes, plants sprayed with 0.5 and 1.0 mM STS produced significantly (P ≤ 0.05) more flowers over a longer duration, and in turn, had larger flower integrals than controls and the 0.125 mM STS treatment. These STS effects on flower numbers and longevity were consistently significant (P ≤ 0.05) in all three genotypes.

**Table 2:** Effect of STS at various dosages on indices of floral development

	Treatment (STS mM)	Age at flowering (days)		Maximum flower count		Days of flower retention		Flower integral	
TMEB204	0	206	a^§^	11	a	12	a	21	a
	0.125	187	a	15	a	14	ab	25	a
	0.25	180	a	27	ab	33	bc	114	ab
	0.5	178	a	54	b	44	c	260	b
	1	188	a	57	b	35	bc	237	b
TMEB419	0	185	a	8	a	12	a	13	a
	0.125	173	a	9	a	18	ab	20	a
	0.25	164	a	24	ab	33	bc	92	ab
	0.5	167	a	55	bc	40	c	242	bc
	1	164	a	66	c	46	c	333	c
IBA980002	0	152	a	4	a	5	a	10	a
	0.125	134	a	26	b	16	b	87	ab
	0.25	129	a	50	bc	40	bc	237	bc
	0.5	138	a	110	c	63	c	677	cd
	1	129	a	83	c	53	c	469	d
All Genotypes	0	181	a	7	a	10	a	15	a
	0.125	167	a	16	ab	16	a	40	a
	0.25	160	a	32	b	35	b	140	b
	0.5	166	a	66	c	46	b	336	c
	1	160	a	68	c	44	b	346	c
ANOVA^¶^									
Treatment main effect		NS		***		***		***	
Genotype effect		***		NS		NS		*	
Block		NS		**		*		**	
Genotype × treatment		NS		·		NS		NS	

The experiment included the genotypes TMSI980002, TMEB419 and TMEB204; data shown are the averages for these genotypes with four replicate blocks

^§^Comparisons between treatments within each genotype with different lowercase letters are significantly (P ≤ 0.05) different using the Tukey HSD multiple range test; based on square root transformation of data

^¶^ANOVA based on a model with STS treatment (T), Genotype (G), Block and GXT interaction effects. Analyses were based on square root transformed data. The symbols ·,*, **, or *** indicate significance at the 0.1, 0.05, 0.01, and 0.001 probability level, respectively. NS indicates no significant difference

In contrast to the effect of STS on floral development, STS did not affect growth of the shoot (leaves and stems), number of storage roots, or storage-root harvest index, as indicated by the absence of significant (P ≤ 0.05) effects on these properties in response to STS treatment, genotype and genotype × treatment interaction (Table 3). The lack of significant differences indicates that STS did not negatively impact plant growth. Harvest index, the ratio of storage root dry weight to whole plant dry weight, was quite high, between 0.52 and 0.58, despite the early growth stage at harvest (140 days after planting).

**Table 3:** Comparison of genotypes and various dosages of STS treatments on total plant dry weight and storage-root harvest index

Genotype	Total plant dry weight (g)		Root count		Harvest Index	
TMEB204	572	a^§^	13	a	0.57	a
TMEB419	579	a	13	a	0.58	a
IBA980002	442	a	11	a	0.51	a
Treatment (STS mM)						
0	563	a	12	a	0.58	a
0.125	541	a	13	a	0.56	a
0.25	489	a	13	a	0.54	a
0.5	563	a	12	a	0.57	a
1	508	a	12	a	0.52	a
ANOVA^¶^						
Treatment main effect	NS		NS		NS	
Genotype effect	*		NS		NS	
Block	NS		**		NS	
Genotype × treatment	NS		NS		NS	

The experiment included the genotypes IBA980002, TMEB419 and TMEB204. ANOVA results are shown for the modelled sources of variation. Genotype values are averages across all STS treatments. Values for STS dosages are averages across all genotypes

^§^Comparisons between genotypes which have different letters are significantly (P ≤ 0.05) different by Tukey’s HSD test

^¶^ANOVA based on a model with STS treatment (T), Genotype (G), Block, and G X T interaction effects. The symbols *, **, or *** indicate significance at the 0.05, 0.01, and 0.001 probability level, respectively. NS indicates no significant difference. There were four replicate blocks

### Ethylene production rate

**Fig. 2:**
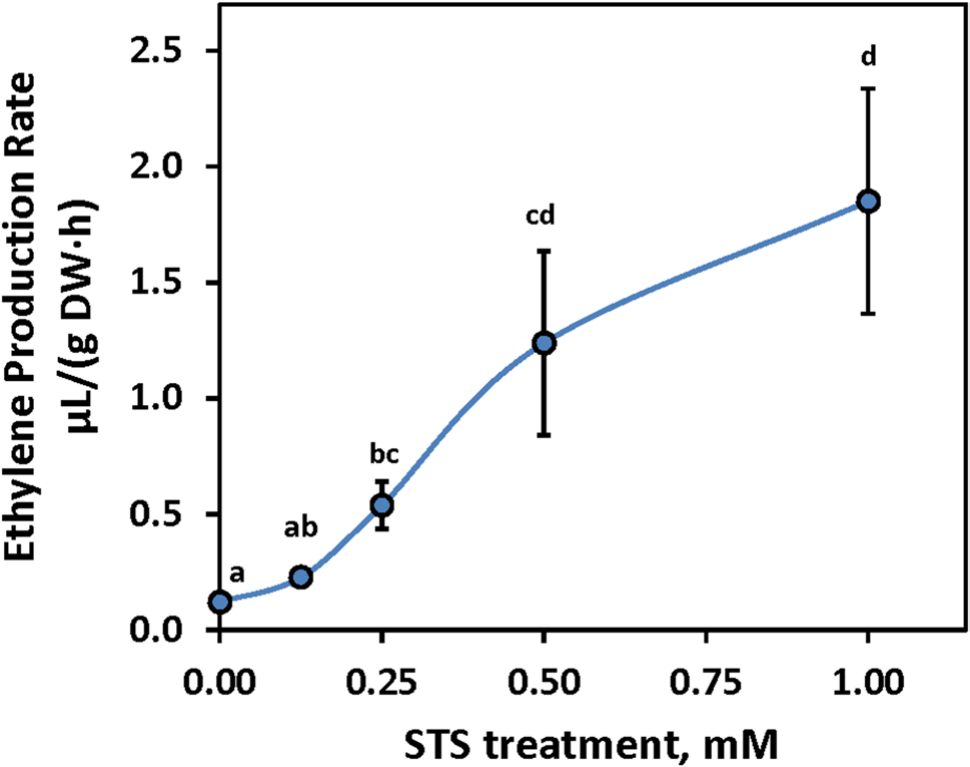
Effect of various dosages of STS on ethylene production in the leaves. Treatments labelled with different lowercase letters were significantly (P ≤ 0.05) different using Tukey’s HSD test on the square root of μL/(g DW h) ethylene. Mean ± SEM are shown

Given past evidence in many plant species that ethylene synthesis is regulated by the ethylene response system via either feedback or feed-forward regulation (Argueso et al. 2007; Atta-Aly et al. 1987; Inaba et al. 2007; Mullins et al. 1999; Nakatsuka et al. 1998), we tested the effect of foliar STS application on ethylene production rate. STS treatments with ≥ 0.25 mM significantly (P ≤ 0.05) increased the rate of leaf ethylene production in proportion to the concentration of STS applied (Fig. 2). This effect indicates that when applied to leaves, STS upregulated ethylene production in a response consistent with interference with feedback inhibition.

### Silver transport in leaves

**Fig. 3:**
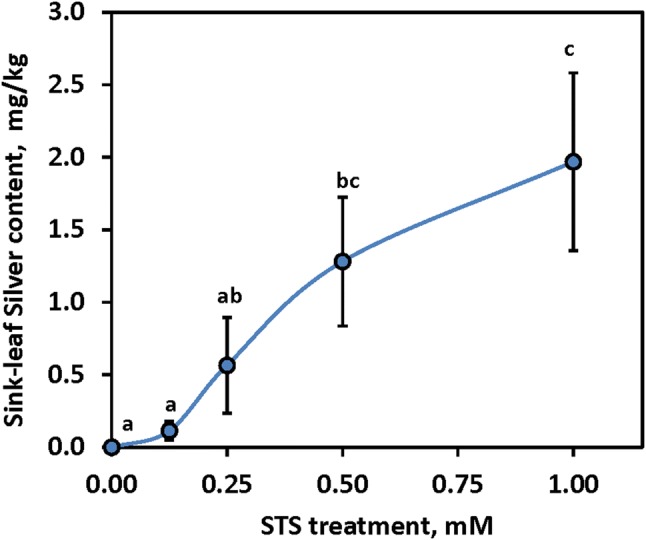
Effect of various doses of STS on quantity of silver in non-sprayed sink leaves at the shoot apex. Treatments labelled with different lowercase letters were significantly (P ≤ 0.05) different using Tukey’s HSD test on data with a square root transformation. Mean ± SEM are shown

While STS was applied to leaves, it is expected that the target tissues for floral effects are the floral organs. To determine whether silver was transported from mature leaves to young non-photosynthetic tissue of the shoot apex, STS was sprayed onto fully-expanded mature leaves and 2 weeks later tissues of the apical meristem region, which was protected from direct spray, was tested for silver content (Fig. 3). Plants sprayed with 1.0 mM STS had significantly more silver accumulation in young sink tissue than plants treated with 0.125 mM STS or controls. Silver accumulation in apical tissue followed a trend similar to the STS dosage response for floral effects (Fig. 1; Table 2), indicating that this method of application delivered silver to the apical region where floral effects were observed.

### STS blocks ethylene effects from ethephon

**Fig. 4:**
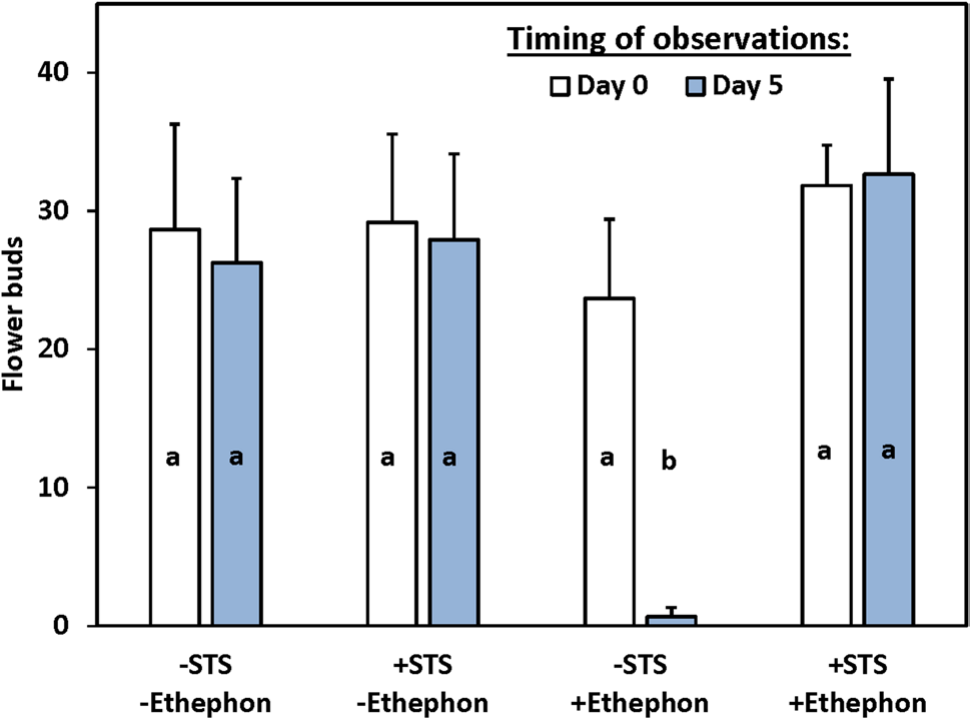
Mean flower buds ± SEM on Day 0 before 500 ppm (w/v) ethephon application (white) and on Day 5 after ethephon application (blue). Plants were pre-treated on Day 0 with (+) or without (−) 0.5 mM STS as a foliar spray, as labelled on the x-axis. Means with different lowercase letters were significantly (P ≤ 0.05) different using Tukey’s HSD test. (Color figure online)

To provide a further test of the hypothesis that STS affects floral development and longevity by blocking the ethylene signaling response, we designed a set of STS and ethephon treatments in cassava plants that had previously developed a set of flower buds without any treatment (Fig. 4). Plants were either pretreated with STS to block ethylene receptors or given a water control treatment, then ethephon was applied to generate ethylene. In this experiment, the control treatments (STS, −ethephon; Fig. 4) had about 28 flower buds per inflorescence and flower production was essentially complete at Day 0, as indicated by the lack of increase in flowers in the controls from Day 0 to Day 5. Treatment with STS did not affect flower numbers (+STS, −ethephon; Fig. 4). In plants not given STS but treated with ethephon, most flower buds senesced and abscised within 5 days of treatment (−STS, +ethephon; Fig. 4). However, in plants treated with 0.5 mM STS, and subsequently treated 2 days later with ethephon (+STS, +ethephon; Fig. 4), flower buds did not senesce and abscise, and the number of flower buds 5 days after ethephon treatment were not significantly (P ≤ 0.05) different from control plants treated only with water. Given that ethephon generates ethylene, these data indicate that the observed STS effects were due to blocking an ethylene response.

### STS benefit to flowering localized to shoot apex

According to our hypothesis, foliar-applied silver is transported to the apical region where it affects flower development. The results in Fig. 3 confirmed that silver is transported from mature leaves to the shoot apex. We next tested direct application of STS to apical tissues (STS-Apex) as an alternative to application of STS to mature leaves (STS-Leaves) as a way to improve flower retention in cassava (Table 4). This study indicated that inflorescence length increased significantly (P ≤ 0.05) with both the STS-Leaves and STS-Apex treatments compared to the control (Table 4). The STS-Leaves treatment had five times more (P ≤ 0.05) flowers than the control, and the STS-Apex treatment averaged 34 flowers compared to 10 in the control, although this difference was not significantly different (P ≤ 0.05). The lack of statistical significance in this case might due to the lack of precision of the flower number data as several individual plants were damaged due to STS phytotoxicity, which increased variability. Both the STS-Leaves and STS-Apex treatments significantly (P ≤ 0.05) increased flower longevity to values more than four times longer than controls (Table 4, days of flower retention). The STS-Apex treatment had significantly (P ≤ 0.05) longer duration than the STS-Leaves treatment. Hence, even though only a small quantity of STS was applied to the apex, the STS-Apex treatment was at least as effective, or more so, than application of a larger quantity to the much larger surface area of the fully expanded, mature leaves (STS-Leaves). These data support the hypothesis that the target tissue for beneficial effects of STS are the tissues of the apex where floral parts are developing.

**Table 4:** Effect of STS treatments applied to the mature leaves (STS-leaves) versus the expanding tissues of the apical region (STS-apex) on inflorescence length, maximum number of flowers + flower buds, and duration of flower development

Treatment	Spray volume (mL)	Inflor. length (cm)	Maximum flower number	Duration (d)
Control (H_2_O)	100	1.4 a	10.0 a	15 a
STS-leaves	100	8.9 b	51.8 b	67 b
STS-apex	10	9.5 b	34.8 a	72 c

Treatments were with 0.25 mM STS sprayed at the indicated volumes; treatments began 1–2 weeks before flower appearance and were applied bi-weekly until 6–7 weeks after flower appearance. Genotype: TMSI980002. Comparisons between treatments which do not have the same letter are significantly (P ≤ 0.05) different using Tukey’s HSD test. Four replicates were used

### STS application to the apical region was most effective when immediately before flower appearance

To test the timing of STS application, we started STS applications at various times of development before flower appearance such that they extended through various stages of flower development. In this experiment, temporal treatments were early, medium, and late (Table 5). On average, early treatments were applied between − 25 and − 4 days from flowering, medium treatments were applied between − 22 and + 1 from flowering, and late treatments were applied between − 15 and + 7 days from flowering (Table 4). Compared to the control treatment, both the medium and late treatments increased (P ≤ 0.05) all measures of flower development: inflorescence length, number of flowers, duration of non-senescent flowers, the number of open flowers, and the flowering integral (Table 5). In the medium and late treatments, the last STS application was after flowers were visible. In contrast, the early treatment did not significantly (P ≤ 0.05) increase flower development by any of the measures. The early treatment began between 24 and 27 days before the actual flowering date (average of 25 days before flowering), and ended between 3 and 6 days before flowering (average of 4 days before flowering). Furthermore, in contrast to treatments which involved spraying 100 mL of STS solution to mature leaves (Table 4), the treatments in Table 5 involved 10 mL of STS solution applied to the apical region. Thus, in this experiment, the early treatment involved application of a small quantity of STS to the pre-flowering apical region where the folded leaves may have intercepted much of the applied solution and shielded the interior where floral development had not yet occurred.

**Table 5:** Effect of STS treatments started either early, medium or late relative to flower bud appearance on the inflorescence length, maximum number of flowers + flower buds, duration of flower development in days (d), peak number of open flowers, and flower development integral

Timing of STS treatment	Time of first app (d)	Time of last app (d)	Inflor. length (cm)	Maximum flower number	Duration (d)	Open Flowers, number	Integral,^†^ count × d
H_2_O control	NA	NA	2.0 a^¶^	27.8 a	21.0 a	0.0 a	214 a
Early	− 25	− 4	6.0 ab	47.8 ab	20.3 ab	7.0 ab	733 a
Medium	− 22	+ 1	12.8 b	86.0 bc	43.3 bc	19.3 b	2054 b
Late	− 15	+ 7	11.5 b	95.0 c	49.0 c	18.3 b	1783 b

For each treatment, the timing of first and last weekly treatment applications (app) to the apical region are shown in days from inflorescence appearance (forking). Negative values indicate before forking. Genotype: TMSI980002

*NA* not applicable

^†^Flower development integral is the area under the curve of flower count with respect to development time

^¶^Comparisons between treatments which do not have the same letter are significantly (P ≤ 0.05) different using Tukey’s HSD test. Four replicates were used

## Discussion

### STS improves several flower developmental processes

A large body of investigation has elucidated the role of ethylene for certain aspects of reproductive organ development. Particular emphasis has been on the role of ethylene in regulating fruit ripening, and tissue senescence associated with fruit softening and formation of an abscission layer in the pedicel (Barry and Giovannoni 2007; Liu et al. 2015; van Doorn 2002; Xie et al. 2013). Previous studies of ethylene effects on flower development have focused on flower senescence and abscission. In flowers, ethylene hastens mature flower senescence and anti-ethylene treatments such as STS and 1-MCP extend the time from flower opening until senescence (Bunya-Atichart et al. 2006; Dar and Tahir 2018; Rice et al. 2013). Consistent with this, in the present study, when cassava plants that had set flowers were treated with exogenous ethephon to generate ethylene, senescence and abscission of flowers was stimulated, whereas pre-treatment with the anti-ethylene agent STS protected flowers from ethylene-induced senescence and abscission (Fig. 4). Furthermore, STS increased the duration of flower production and retention from 6 days in controls to 33 days with 0.5 mM STS (Table 1), and from 10 days in controls to 44 days with 1.0 mM STS in the dosage experiment (Table 2). These findings indicate that cassava flower longevity is limited due to its sensitivity to ethylene, and by blocking the ethylene response, flower bud development into mature flowers is improved.

Few studies have examined the role of ethylene in early inflorescence and early flower bud development (Cerveny and Miller 2010). In the current work, STS was not only effective in extending the longevity of flowers, it also prolonged flower bud formation such that there was an increase in the number of flowers formed. This indicates that cassava’s poor ability to produce flowers is due, in part, to ethylene inhibition of inflorescence development, and in turn flower formation. In cassava, only a few short-lived flowers are produced at the first tier of inflorescence formation (Fig. 1a and b; Diebiru et al. 2016). STS substantially increased the number of flowers from 4 to 7 in controls, to between 46 and 68 in STS-treated plants (Tables 1, 2, respectively). Thus, the current work indicates that STS improved flowering over several phases of development ranging from sustained formation of inflorescences and production of flowers, to greater flower longevity such that flowers matured normally and did not prematurely abort or abscise.

In contrast to the effects of STS in sustaining floral development, flower initiation was not hastened by STS in cassava (Tables 1, 2). This differs from studies in other plant species where ethylene acts with gibberellin or auxin to affect the timing of flower initiation (Achard et al. 2007; Frankowski et al. 2014). Also, STS did not affect the development or partitioning of biomass production as indicated by the lack of effects on storage root numbers and harvest index (Table 3). In some plant systems, ethylene has a role in regulating vegetative (Dubois et al. 2018) or root development (Lewis and Muday 2013; Pankomera et al. 2016), though reports of ethylene effects on storage root development are rare.

### STS benefit is via blocking ethylene responses in the shoot apical region

Our findings provide several lines of evidence indicating that STS benefits floral development by blocking an ethylene response in the shoot apex where inflorescence and flower development is located: STS application improved inflorescence and flower development and increased flower duration (Tables 1, 2); mature leaves responded to STS by increasing their ethylene production (Fig. 2); silver applied to mature leaves was transported to apical tissues (Fig. 3); STS prevented flower abscission in response to ethylene generated via ethephon treatment, (Fig. 5); and STS application directly to the apical tissues was as effective as spraying the mature leaves, even though the quantity of silver applied was one tenth as much (Tables 4, 5). These findings are consistent with studies which have indicated that STS is capable of transport through the phloem and xylem vascular systems (Beyer 1976; Veen and van de Geijn 1978). The target tissue for the observed effects on floral development are consistent with findings that indicate ethylene elicits flower senescence via direct signaling in floral tissues (Serek et al. 2006, 2015).

The STS dose response appeared to saturate at about 0.5 mM STS (Table 2), which is consistent with the hypothesis that there is a saturation point at which all the ethylene receptors are blocked and there is enough STS available to block any newly developed ethylene receptors (Beyer 1976). Above this level no additional benefit was detected. When STS was applied to mature leaves, silver was detected in newly developed leaves that had not been treated with STS; furthermore, as the treatment concentration increased, the level of silver in newly formed leaves increased correspondingly (Fig. 3). The presence of silver ions in unsprayed leaves indicates that STS is absorbed and transported in the plant for at least 2 weeks. Previous studies have indicated that silver ion is relatively non-mobile within plants (Kofranek and Paul 1975). The positively charged silver ions bind to the anionic surfaces on xylem vessels thereby interfering with its uptake and transport processes (Veen and van de Geijn 1978). However, the mobility and rate of transport of silver ion is improved by complexing it with thiosulfate (Veen and van de Geijn 1978; Veen 1983). In fact, within the xylem, silver ion complexed as STS moves much more freely compared to silver ion applied as silver nitrate (Veen and Van de Geijn 1978).

The current results suggest that the target tissue for favorable effects of STS are in the shoot apical region where floral initiation and inflorescence development take place. Localization of STS application to the apical region was as effective as general foliar spray to the canopy of leaves even though the quantity of STS applied was one tenth as much (Table 4). The ability to apply less STS in this way is potentially valuable as it lessens the chance of incurring STS phytotoxicity, which was observed in some of our studies, as noted above for the experiment reported in Table 4. Such phytotoxicity is known to occur in many plant systems, requiring judicious choice of STS concentration (Hoyer 1998; Serik et al. 2015). Lowering the amount of silver in the spray also decreases the amount of residual silver introduced into plant debris and soil. However, such residues are not likely to affect cassava consumers because the intended use of STS is in breeder’s nurseries, involving relatively small plant numbers, where flowering is needed to make crosses at the early phase of a breeding cycle, not in fields of cassava for storage-root production where plants are vegetatively propagated by stem cuttings.

The stimulation of cassava leaf ethylene synthesis by STS (Fig. 2) is consistent with ethylene production in the cassava leaf system being controlled by negative feedback inhibition (Argueso et al. 2007). By blocking the ethylene receptors, STS releases inhibition caused by downstream ethylene responses, including feedback inhibition of ACC synthesis and/or ACC oxidase, thereby increasing the rate of ethylene synthesis. This finding indicates that cassava leaves regulate ethylene synthesis in a way similar to that found in other plant systems in which there is feedback inhibition of ethylene synthesis, such as young pre-climacteric (green) tomato fruits (Nakatsuka et al. 1998, Atta-Aly et al. 1987), preclimacteric banana fruit (Inaba et al. 2007), non-climacteric citrus fruit (Mullins et al. 1999), and non-climacteric strawberry fruit (Atta-Aly et al. 2000). Hence, by blocking ethylene effects, STS improved flower development and longevity (Table 1 and 2) despite the treatment stimulating ethylene production in leaves.

## Conclusions

The current studies indicate that anti-ethylene STS treatments substantially increase the prolificacy and longevity of flower production in cassava. These findings complement a large body of work in other species that has elucidated ethylene roles in reproductive development during fruit formation and flower senescence. Our work shows that in cassava, ethylene exerts negative effects even at early stages of inflorescence and floral development. Anti-ethylene treatment with STS was able to prevent abortion of inflorescences and flowers such that large numbers of mature flowers were produced with extended longevity. Our studies show that the target tissues for the favorable effects of STS are the apical regions of cassava plants. Based on this work, we recommend applications of 0.25 to 0.5 mM STS to the shoot apical region using four weekly applications beginning about 2–3 weeks before inflorescence appearance. This work has the potential to improve methods for regulating cassava flower development in breeding nurseries and thereby synchronize flowering of desired parents and enable the production of abundant progeny of desired crosses. With these improved methods of regulating flowering, breeding programs will have the potential to increase the rate of genetic improvement and be better able to deliver cultivars that are needed by cassava farmers.

## Electronic supplementary material

Below is the link to the electronic supplementary material.

Supplementary Table S1. PGR 1: The effect of gibberellic acid (GA3), paclobutrazol and *p*-chlorophenoxyisobutyric acid (PCIB) compared to that of a non-treated control on age at first flowering and plant height. This experiment consisted of 4 genotypes TMS1980002, Nase 3, TME 204, and TME 419 replicated 4, 5, 5 and 6 times respectively. PGR 2: The effect of 6-benzyl adenine (BA), jasmonic acid (JA), salicylic acid (SA) and silver thiosulfate (STS) compared to that of a non-treated control. This experiment consisted of four genotypes TMS1980002, Nase 3, TME 204, and TME 419 replicated four times each for total of 80 experimental units. PGR 3: The effect of abscisic acid (ABA), aminoethoxy vinyl glycine (AVG), and silver thiosulfate (STS) compared to that of a non-treated control. This experiment consisted of four genotypes TMS1980002, Nase 3, TME 204, and TME 419 replicated four times. PGR 4: The effect of 1-methylcyclopropene (1-MCP) compared to that of a non-treated control on flowering at tier 1 branching. This experiment consisted of two genotypes, TMS1980002 and Nase 3, replicated four times. PGR 5: The effect of 1-methylcyclopropene (1-MCP) applied as a foliar spray compared to that of a non-treated control on flowering at tier 1 branching. This experiment was replicated four times. Supplementary material 1 (XLSX 15 kb)

## Abbreviations

DAP: Days after planting
PGR: Plant growth regulator
STS: Silver thiosulfate
